# Affective Temperaments and Anger in Patients with Tinnitus and High-Frequency Sensorineural Hearing Loss: A Pilot Cross-Sectional Study

**DOI:** 10.3390/medicina62020340

**Published:** 2026-02-07

**Authors:** Daniele Portelli, Clara Lombardo, Sabrina Loteta, Francesco Ciodaro, Cristina Bartolotta, Cosimo Galletti, Carmela Mento, Angela Alibrandi, Giuseppe Alberti

**Affiliations:** 1 Unit of Otorhinolaryngology, Department of Adult and Development Age Human Pathology “Gaetano Barresi”, University of Messina, 98122 Messina, Italy; daniele.portelli09@gmail.com (D.P.); lotetas@unime.it (S.L.); dottfciodaro@alice.it (F.C.); galberti@unime.it (G.A.); 2 Department of “Scienze della Salute”, University of “Magna Graecia”, 88100 Catanzaro, Italy; 3 Department of Biomedical and Dental Sciences and Morphofunctional Imaging, University of Messina, 98122 Messina, Italy; cristina.bartolotta@tim.it (C.B.); cmento@unime.it (C.M.); 4 Faculty of Medicine and Surgery, Kore University of Enna, 94100 Enna, Italy; cosimo.galletti@unikore.it; 5 Unit of Statistical and Mathematical Sciences, Department of Economics, University of Messina, 98122 Messina, Italy; aalibrandi@unime.it

**Keywords:** tinnitus, affective temperaments, THI, TFI, STAXI-2, hearing loss, high-frequency hearing loss, ski slope

## Abstract

*Background and Objectives*: Chronic tinnitus in patients with bilateral sensorineural hearing loss (SNHL) is frequently linked to psychological distress and affective temperament traits. This study examined audiological characteristics, psychological profiles, and their interrelations in adults with tinnitus. *Materials and Methods*: In this cross-sectional single-centre study, 38 adults with chronic tinnitus and bilateral SNHL underwent otoscopy, tympanometry, and pure-tone audiometry. Participants were classified into two audiometric groups: high-frequency gently sloping (N = 19) and ski-slope hearing loss (N = 19). The audiological and psychological assessment included the THI, TFI, TEMPS-A, and STAXI-2. Groups were compared using non-parametric statistics (Mann–Whitney U, Chi-square). Relationships between anger and psychological variables were examined using Spearman correlations. *Results*: Females were more frequently represented in the gently sloping group (*p* = 0.044) and showed greater quality-of-life impairment (*p* = 0.045) and lower hyperthymic scores (*p* = 0.004). Patients with gently sloping loss had longer tinnitus duration (*p* = 0.026), while cyclothymic temperament was higher in the ski-slope group (*p* = 0.013). THI scores differed significantly between audiometric groups (*p* = 0.011). State and trait anger were strongly associated with several affective temperaments, and sleep disturbance correlated with both anger and anxious temperament. THI correlated positively with anxious temperament and sleep impairment. *Conclusions*: Audiometric phenotype, affective temperament, and anger-related traits are closely intertwined with tinnitus severity and its psychological burden. These findings support the need for multidisciplinary assessment in the management of tinnitus.

## 1. Introduction

Tinnitus is defined as the conscious perception of sound in the absence of external acoustic stimuli and affects approximately 10–15% of the adult population worldwide, with 1–2% reporting a level of distress that markedly compromises quality of life as well as social and occupational functioning [1]. In most cases, tinnitus is associated with sensorineural hearing loss (SNHL) and, especially in older adults, can entail substantial psychological and emotional repercussions.

Recent findings from large perspective studies and systematic meta-analyses have strengthened the evidence for a bidirectional relationship among tinnitus, SNHL, and mental health disorders. Tinnitus is linked to a 30–48% increase in the risk of anxiety and depression, while insomnia, chronic stress, and suicidal behaviours are more frequent in affected patients [2,3]. Studies have shown a relationship between different affective temperaments and physical illnesses, such as autoimmune diseases or chronic pain disorders [4,5]. Patients with a predominantly cyclothymic affective temperament presented more frequently with diabetes [6], while those with high scores in the hyperthymic temperament had a higher incidence of hypertension [7]. We believe that people with tinnitus possess a biological predisposition to the development of this condition, which could manifest through a specific affective reaction mode, namely, affective temperament.

According to Akiskal’s model of affective temperaments, individuals may exhibit depressive, cyclothymic, hyperthymic, anxious or irritable dispositions, which in turn shape their response to stress and illness [8]. Affective temperaments are associated with greater distress and reduced coping capacity in individuals with tinnitus [9]. The study of Trifunovic et al. (2020) showed that patients with chronic tinnitus predominantly had an anxious affective temperament (anxious-somatic was dominant in people without hearing loss and in men, while anxious-cognitive was dominant in people with significant hearing loss and in women) [10]. Also, Langguth et al. (2013) demonstrated that greater neuroticism—which conceptually overlaps with depressive and anxious temperaments—significantly predicts tinnitus-related distress [11]. Similarly, Bartels et al. (2010) found that cyclothymic and anxious traits are associated with a greater subjective impact of tinnitus, probably mediated by increased limbic activation and altered prefrontal regulation of emotional responses [12]. In particular, affective temperaments characterized by high emotional reactivity may predispose to higher levels of trait anger and more intense state anger responses in the face of frustration and uncontrollability associated with tinnitus. Anger is an emotional state that can range from mild irritation to intense fury and rage. When a person tends to feel angry often, very strongly, and for a long time, this is called trait anger, a stable part of their personality. The expression of anger refers to how anger is managed, whether it is expressed outwardly, held in, or controlled [13]. A study of 100 patients with chronic tinnitus found high levels of “emotional excitability” and “inhibited aggression”, elements that can be traced back to trait anger, which could contribute significantly to symptom-related distress. In addition, the subjective experience of tinnitus, perceived as intrusive and uncontrollable, increases the intensity of emotional distress. These emotional dimensions tend to intertwine personal predisposition (trait anger), in combination with transient states of anger (state anger), amplifies the negative perception of tinnitus [14].

Cross-sectional studies further reveal significant sex differences: women with tinnitus report higher levels of emotional distress, insomnia, and depressive symptoms than men, suggesting a complex interaction of hormonal, cognitive, and sociocultural factors in shaping the psychological response to tinnitus [12].

From an audiological standpoint, the clinical profile of tinnitus may be influenced by different audiometric curves [15]. However, their relationship with patients’ psychological patterns remains under-explored. An integrated characterization of the psychological correlates of tinnitus—and their interaction with demographic and audiological variables—is therefore essential for developing highly individualized therapeutic protocols [16].

The present study aims to examine the role of affective temperaments and anger in patients with tinnitus and sensorineural hearing loss. In particular, a comparative analysis was carried out considering gender (Male/Female) and the morphology of the hearing threshold (high-frequency gently sloping hearing loss vs. ski-slope hearing loss). Beyond the presence and severity of hearing loss, the morphology of the audiometric curve represents a clinically meaningful dimension that may shape the subjective experience of tinnitus. Gently sloping and ski-slope high-frequency hearing losses differ not only in their acoustic consequences but also in the predictability, abruptness, and perceived controllability of auditory impairment. A ski-slope configuration, characterized by a sharp high-frequency drop with relative preservation of low frequencies, may generate a greater discrepancy between expected and actual auditory performance, potentially increasing frustration and emotional reactivity. In contrast, a gently sloping loss reflects a more gradual sensory decline that may allow progressive adaptation over time [17]. Furthermore, personality traits may modulate how individuals perceive and emotionally respond to both hearing loss and tinnitus, potentially amplifying or mitigating the associated distress. This grouping therefore allows for a more nuanced analysis of the interplay between hearing loss morphology and personality-related perceptual and emotional responses.

In this context, examining anger-related dimensions together with audiometric morphology represents a novel approach. While previous studies have investigated affective temperaments or general psychological distress in tinnitus, the integration of state and trait anger (STAXI-2) with distinct audiometric phenotypes has received little attention. Anger-related traits are particularly relevant in tinnitus, a condition frequently associated with perceived loss of control and persistent frustration. By comparing patients with gently sloping and ski-slope hearing loss, the present study aims to explore whether different auditory phenotypes are associated with distinct anger profiles and temperamental characteristics, thereby contributing to a more refined psychoaudiological characterization of tinnitus. We formulated the following hypotheses: (1) patients with a ski-slope configuration would show higher tinnitus-related handicap (THI) than those with a high-frequency gently sloping hearing loss; (2) higher levels of state and/or trait anger may be associated with temperaments characterised by greater emotional reactivity (cyclothymic, depressive, irritable); and (3) anxious temperament and sleep disturbances (TFI-Sleep) may be positively associated with tinnitus-related handicap.

## 2. Materials and Methods

### 2.1. Study Design, Setting and Participants

The study was a pilot cross-sectional, observational single-centre study. A total of 38 adults with chronic tinnitus and bilateral SNHL (M: 24, F: 14) were enrolled in this study. Inclusion criteria were an age of at least 18 years, tinnitus for at least six months, and documented binaural SNHL, which was clinically verified using pure-tone audiometry. Exclusion criteria were as follows: past neurological and/or psychiatric illness under pharmacological treatment, use of hearing aids at the time of evaluation, and incomplete questionnaire answers. The study adhered to the principles of the Declaration of Helsinki and was approved by the Ethics Committee at the University Hospital of Messina (Protocol 97/24, 19 July 2024). All participants provided consent for the application of hearing aids, audiological testing, and the use of their data during counselling sessions.

### 2.2. Audiological Assessment

In the initial phase, otoscopic examination was performed using a microscope, along with tympanometry, to rule out any pathology of the external and middle ear. This was followed by conventional pure-tone audiometry conducted in a soundproof booth, using TDH-39 headphones for air conduction and a B71 bone vibrator for bone conduction. The standard pure tone average (PTA) was then calculated by averaging the thresholds at the central frequencies of 0.5, 1, and 2 kHz. The patients were divided into two groups based on hearing loss morphology: high-frequency gently sloping hearing loss (HFGS; *n* = 19) and ski-slope hearing loss (SL; *n* = 19).

High-frequency gently sloping hearing loss was defined by an audiometric curve where the dB HL gap between various frequencies (125, 250, 500, 1000, 2000, 4000, 8000) was 30 dB HL or less. Ski-slope hearing loss was defined according to the criteria set by Schuurbiers et al. (2017): hearing thresholds of 25 dB or less at 250 Hz, 80 dB or more at 4 kHz, and a decrease of 70 dB or more between 500 Hz and 4 kHz, or 40 dB or more between 250 Hz and 1 kHz, or between 500 Hz and 2 kHz [18].

### 2.3. Psychological and Tinnitus Assessment

Participants completed a battery of standardized self-report questionnaires and demographic variables (age, gender, education level, profession) and tinnitus duration (years) were also collected.

- **Tinnitus Handicap Inventory (THI):** Used to assess tinnitus-related distress and to evaluate the impact of tinnitus on a patient’s daily life. It comprises 25 items grouped into three subscales—functional, emotional, and catastrophic—addressing the influence of tinnitus on concentration, sleep, emotional well-being, and social interactions [19]. Scoring is based on patients’ responses to each of the 25 items, with three possible answers: “yes” (4 points), “sometimes” (2 points), and “no” (0 points). The total score, ranging from 0 to 100, is obtained by summing the points for all items. Higher scores indicate greater tinnitus-related distress, and is classified into five severity levels: slight (0–16), mild (18–36), moderate (38–56), severe (58–76), and catastrophic (78–100) [19].
- **Tinnitus Functional Index (TFI):** An instrument used to assess the impact of tinnitus on an individual’s daily life. It measures in detail how much and in which areas the symptom of tinnitus (ringing, buzzing or other sounds perceived in the ears or head) creates discomfort or limits activities. It consists of 25 questions, each rated on an 11-point Likert scale. Patients answer each question based on how they have felt in the last week. The TFI includes eight subscales: Intrusiveness (questions 1–3), Sense of Control (questions 4–6), Cognitive (questions 7–9), Sleep (questions 10–12), Auditory (questions 13–15), Relaxation (questions 16–18), Quality of Life (questions 19–22) and Emotions (questions 23–25). The total score ranges from 0 to 100 and is divided into five levels of clinical severity: no problem (0–17), mild problem (18–31), moderate problem (32–53), severe problem (54–72), very severe problem (73–100). In addition to the overall score, the scores of the individual subscales can also be calculated. A decrease of 13 points in the TFI score is considered clinically significant and indicates a reduction in distress [19,20,21].
- **Temperament Evaluation of Memphis, Pisa, Paris, and San Diego—Auto-questionnaire (TEMPS-A).** The short version of this questionnaire was considered more suitable for large-scale use than the original version, which included 110 questions. This short version consists of 39 questions with a dichotomous response (Yes/No) and allows for the assessment of affective temperaments on five subscales: cyclothymic (questions 1–12), depressive (questions 13–20), irritable (questions 21–28), hyperthymic (questions 29–36) and anxious (questions 37–39). These temperaments are considered subclinical traits that may increase vulnerability to specific mood disorders. The internal consistency of the TEMPS-A subscales, as measured by Cronbach’s α, was good for all dimensions (>0.70) [22,23].
- **State-Trait Anger Expression Inventory (STAXI-2): State–Trait Anger Expression Inventory (STAXI-2)** is a comprehensive instrument designed to assess anger across multiple dimensions: the intensity of anger as an emotional state (state anger; SANG), the individual’s predisposition to experience anger as a personality trait (trait anger; TANG), as well as the internal and external expression of anger and anger control. The state anger subscale measures current, situational experiences of anger, whereas the trait anger subscale assesses an individual’s general tendency to experience anger and related characteristics over time. The inventory consists of 57 items rated on a 4-point Likert scale, which evaluates the intensity, expression, suppression, and control of anger [24]. Within the STAXI-2, state anger (SANG) and trait anger (TANG) subscales were considered. In the present study, the analysis was limited to the State Anger (SANG) and Trait Anger (TANG) subscales, as these dimensions respectively reflect the situational experience of anger and the stable individual tendency to respond with anger. These constructs are particularly relevant to chronic tinnitus, a condition frequently characterized by feelings of frustration, loss of control, and emotional distress. The remaining STAXI-2 dimensions, related to anger expression and anger control, were not included in order to reduce participant burden and to limit the number of statistical comparisons in this exploratory sample.

### 2.4. Statistical Analysis

Data analysis was performed using IBM SPSS Statistics for Windows, Version 26.0 (IBM Corp., Armonk, NY, USA). Quantitative data were presented as medians and interquartile ranges (IQRs, Q1–Q3). The categorical data were presented as proportions and percentages. The critical psychological and audiological variables were found to have non-normal distributions as assessed by the Kolmogorov–Smirnov test and as such, non-parametric statistics was used in all analyses. Between-groups comparisons of continuous variables (age, tinnitus duration, THI scores, psychological variables, TFI) by gender as well as by audiometric group were performed using the Mann–Whitney U test. Categorical comparisons (e.g., gender distribution across audiometric group) were made using the Chi-square or Fisher’s exact test as applicable. Spearman’s rank order correlation coefficients were calculated in order to examine relationships among psychological variables, age, tinnitus duration, THI and, TFI.

No a priori power analysis was performed, as the study was designed as an exploratory pilot investigation conducted in a single-centre clinical setting. Given the exploratory nature of the study and the relatively small sample size, no formal correction for multiple comparisons was applied. Furthermore, no correction was deemed necessary as comparisons were made between two independent and non-overlapping grouping variables, without cross-over or factorial designs. Statistical findings were interpreted with caution, with greater emphasis placed on the direction and consistency of effects, as well as on their clinical plausibility, rather than on isolated *p*-values. A 2-tailed *p* < 0.05 was considered statistically significant. Box plots were constructed to visually show the differences between the groups.

## 3. Results

### 3.1. Participant Characteristics

A total of 38 patients (24 males [63.2%], 14 females [36.8%]) were enrolled in the study. The participants were predominantly university graduates (68.4%), while the others held high school diplomas (31.6%). The most common professions were pensioner (36.8%) and entrepreneur (15.8%). There was an equal number of audiometric profiles with high-frequency gently sloping hearing loss (*n* = 19, 50%) and ski-slope hearing loss (*n* = 19, 50%) (Table 1).

The mean age was 66.7 (standard deviation, SD ± 12.45) and the mean duration of tinnitus was 4.49 (standard deviation, SD ± 2.43). Table 2 shows the numerical variables present in the study expressed as median and interquartile range (IQR) (Table 2).

### 3.2. Gender-Based Comparisons

There was a significant relationship between sex and audiometric phenotype. Females were more frequently represented in the high-frequency gently sloping hearing loss group (71.4%) compared to males (37.5%) (χ^2^ = 4.07, *p* = 0.044) (Table 3).

There were no differences between males and females for tinnitus duration (*p* = 0.501) and THI score (*p* = 0.544). Nevertheless, females had significantly more impaired quality of life (*p* = 0.045) and lower scores in hyperthymic temperament (*p* = 0.004) (Table 4).

### 3.3. Audiometric Group Comparisons

Subjects with high-frequency gently sloping hearing loss had a longer tinnitus duration compared to subjects with ski-slope loss (*p* = 0.026).

The cyclothymic temperament was significantly more in the ski-slope group (*p* = 0.013).

There were differences in THI scores between the groups (*p* = 0.011) (Table 4) (boxplot available in Supplementary Materials Figure S1).

### 3.4. Correlation Analyses

Correlation analyses revealed significant associations among the study variables. State anger correlated positively with cyclothymic (r = 0.495, *p* = 0.002), depressive (r = 0.615, *p* < 0.0001), irritable (r = 0.664, *p* < 0.0001), and hyperthymic (r = 0.339, *p* = 0.037), temperaments. Meanwhile, trait anger correlated positively with cyclothymic (r = 0.659, *p* < 0.0001), depressive (r = 0.722, *p* < 0.0001), and irritable (r = 0.636, *p* < 0.0001) temperaments. A direct correlation also emerged between the TFI subscale “Sleep” and state anger (r = 0.53, *p* = 0.001), as well as with anxious temperament (r = 0.340, *p* = 0.037).

Finally, tinnitus handicap index (THI) scores were positively correlated with anxious temperament (r = 0.345, *p* = 0.034) and sleep disturbance (r = 0.355, *p* = 0.029) (see Table S1 in Supplementary Materials).

## 4. Discussion

The results of the present study highlight a significant relationship between sex and audiometric phenotype in patients with tinnitus and sensorineural hearing loss. Given the pilot cross-sectional design, the observed relationships should be interpreted as associations and do not allow causal inferences regarding whether temperamental traits and anger contribute to tinnitus distress or represent consequences of chronic symptom burden.

Women were more frequently represented in the high-frequency gently sloping configuration group (71.4%) compared with men (37.5%). This finding is consistent with the literature, which documents a higher prevalence of tinnitus and self-reported hearing loss among women, particularly in older age groups [25,26].

Although no significant sex differences emerged with respect to tinnitus duration, women reported a significantly poorer quality of life compared with men. This result confirms previous evidence describing greater emotional vulnerability in women with regard to tinnitus and a higher incidence of psychological distress [27,28].

### 4.1. Audiometric Phenotypes and Affective Temperaments

In line with previous literature highlighting the role of affective temperaments in modulating individual vulnerability to chronic distress [8,10,29], the lower expression of hyperthymic temperament observed in women may represent a relevant factor in understanding their reduced coping capacity and increased susceptibility to psychological burden.

The analysis of audiometric subgroups revealed clinically meaningful differences. Patients with high-frequency gently sloping hearing loss reported a significantly longer duration of tinnitus, suggesting a more gradual and insidious clinical course. In contrast, individuals with a ski-slope configuration showed significantly higher cyclothymic temperament scores, a profile characterized by emotional instability and mood fluctuations, which has previously been associated with greater tinnitus-related distress [10].

Moreover, patients with a ski-slope configuration exhibited significantly higher THI scores compared with those with high-frequency gently sloping hearing loss, indicating a greater subjective impact of tinnitus. This finding may be explained by the presence of more emotionally reactive temperamental traits within this subgroup, potentially increasing the sensitivity to auditory symptoms and reducing tolerance to chronic tinnitus perception.

### 4.2. State and Trait Anger and Affective Temperaments

Correlation analyses showed that state anger was associated with cyclothymic, depressive, irritable, and hyperthymic temperaments, whereas trait anger correlated with cyclothymic, depressive, and irritable temperaments. These temperaments, which are typically associated with heightened emotional reactivity, anxiety, and stress [22], may amplify the perception of tinnitus and the related distress. Our results are consistent with previous evidence linking emotional dysregulation to psychosomatic disorders and chronic tinnitus [30,31].

### 4.3. Sleep, Psychological Distress, and Tinnitus

Another noteworthy finding concerns the correlation between the “Sleep” subscale of the TFI, state anger, and the anxious temperament. Sleep disturbances are among the most common comorbidities in tinnitus patients [32] and contribute to the maintenance of distress by lowering the tolerance threshold and amplifying emotional reactivity. The anxious temperament may act as a mediator between affective vulnerability and sleep dysregulation, delineating a vicious cycle of insomnia, emotional hyperarousal, and worsening tinnitus symptomatology [33,34,35].

### 4.4. Psychological Determinants and THI

Finally, the association between Tinnitus Handicap Inventory (THI) scores and anxious temperament further supports the role of psychological and temperamental factors in shaping the subjective perception of tinnitus-related handicap. Perceived tinnitus severity does not depend solely on audiological parameters but is strongly modulated by the individual’s emotional response and personality structure [12,35].

Assessing personality traits in patients with tinnitus may help explain the marked variability in tinnitus-related distress observed among individuals with comparable audiometric profiles. Identifying distinct psychological phenotypes may clarify differences in emotional burden and coping strategies and may guide the selection of more appropriate therapeutic pathways, for example, prioritizing cognitive–behavioural interventions when maladaptive emotional or cognitive patterns are predominant, or sound-based approaches when the auditory component plays a more central role.

Audiological phenotype differences should be considered in personalized management programs. It is well known that one of the strategies for managing tinnitus involves the use of sound amplification systems or sound stimuli, such as noises or fractal tones [36]. The primary aim is to divert the patient’s attention away from tinnitus perception. Since tinnitus is most commonly associated with hearing loss, a wide variety of digital hearing aids—differing in both form and function—are now available [37,38,39]. Selecting the most appropriate hearing aid based not only on audiological patterns but also on individual patient characteristics has become increasingly important [40]. This highlights the need to investigate tinnitus phenotypes in order to tailor therapeutic strategies to each individual. Integrating psychometric assessment into the clinical evaluation process therefore supports a more individualized and targeted approach to tinnitus management, aligning therapeutic strategies with both the audiological and psychological profiles of each patient.

From a clinical point of view, the results of the study suggest the usefulness of an integrated assessment of patients with tinnitus that includes, in addition to audiological parameters, their temperamental and emotional profile. The identification of specific temperamental traits and high levels of state or trait anger could allow for the early identification of patients at greater risk of psychological distress. In this context, the classification of the audiometric phenotype, which is associated with individual psychological characteristics, can support the choice of more targeted therapeutic interventions, favouring a personalised approach. The integration or selective use of audiological treatments and psychological interventions—either alone or in combination—may enhance the overall effectiveness of tinnitus management, allowing therapeutic strategies to be tailored to the individual patient and thereby improving quality of life.

### 4.5. Limitation

Our study has several limitations that should be considered when interpreting the results. First, the relatively small sample size, which is related to the pilot cross-sectional design, limits the generalizability of the findings and their extension to the broader tinnitus population. Secondly, the single-centre design of the study may reflect clinical and organisational characteristics specific to the recruitment context, limiting the extension of the results to other clinical settings. Furthermore, the cross-sectional nature of the study does not allow causal relationships to be established between temperamental characteristics, audiological variables and tinnitus severity. It is therefore not possible to determine whether specific personality traits are a predisposing factor or a consequence of chronic tinnitus experience. Finally, the use of self-report instruments, although validated, could introduce a potential response bias related to the patient’s subjective perception. However, this limitation was partially mitigated using standardised questionnaires widely used in other studies. Future studies with larger samples, multicentre designs and longitudinal approaches are needed to confirm and expand on the findings, as well as to explore the temporal evolution of interactions between audiological profile, affective temperament and tinnitus distress.

## 5. Conclusions

The present findings highlight the complex relationship between affective temperaments, anger-related traits, and audiometric phenotype in shaping tinnitus-related distress. Differences in emotional vulnerability among patient subgroups underline the importance of an integrated clinical approach that extends beyond audiological assessment alone. Incorporating psychological profiling into routine tinnitus evaluation may represent an innovative approach to the management of this condition. Systematic use of these psychometric tools could help identify patients at higher risk of distress and support a more personalized selection of therapeutic strategies.

Multidisciplinary interventions combining audiological rehabilitation with psychological support may therefore represent an effective approach to improving the quality of life in individuals with chronic tinnitus.

## Figures and Tables

**Table 1:** Categorical variables of patients included in the study.

	Frequency	Percentile (%)
Sex	38	100
Male	24	63.2
Female	14	36.8
Educational qualification	38	100
High school graduate	12	31.6
University graduate	26	68.4
Hearing loss profile	38	100
High-frequency gently sloping hearing loss	19	50.0
Ski-slope	19	50.0

**Table 2:** Numerical variables expressed as median and interquartile range (IQR).

	Percentile		
	25°	50°	75°
Age	60.50	70.50	74.00
Tinnitus duration	3.00	4.00	5.63
THI	27.00	47.00	68.00
Right PTA	40.00	46.67	53.75
Left PTA	41.67	45.84	55.00
SANG	44.00	51.00	58.50
TANG	43.50	55.00	64.00
Cyclothymic	2.00	5.00	7.25
Depressive	0.75	4.00	6.00
Irritable	0.00	2.00	3.00
Hyperthymic	2.75	4.50	6.00
Anxious	0.00	1.00	2.25
TFI total score	45.10	54.50	65.90
TFI Intrusiveness	52.50	63.33	70.00
TFI Sense of Control	40.00	56.67	70.00
TFI Cognitive	46.67	60.00	70.00
TFI Sleep	25.83	50.00	63.33
TFI Auditory	49.17	61.67	70.00
TFI Relaxation	42.50	53.33	67.50
TFI Quality of life	37.50	51.25	68.13
TFI Emotional	46.67	56.67	66.67

THI, tinnitus handicap inventory; TFI, tinnitus functional index; PTA, pure tone average; SANG, state-anger; TANG, trait-anger

**Table 3:** Contingency table of hearing loss type and gender.

		Sex		
		M	F	Total
Hearing loss	HFGS	9	10	19
		37.5%	71.4%	50.0%
	SL	15	4	19
		62.5%	28.6%	50.0%
Total		24	14	38
		100.0%	100.0%	100.0%
Pearson chi-square test				*p*-value
				0.044 *

HFGS, high-frequency gently sloping (N = 19); SL, ski-slope (N = 19); * statistically significant.

**Table 4:** Comparison of audiological and psychological variables by sex and by audiometric phenotype (HFGS vs. SL). Data are reported as mean ± SD; *p*-values from Mann–Whitney U test (continuous variables).

	Sex	Mean	SD	*p*-Value	Hearing Loss	Mean	SD	*p*-Value
Age	F	67.57	11.35	0.897	HFGS	68.16	11.07	0.371
	M	66.21	13.26		SL	65.26	13.85	
Tinnitus duration (years)	F	4.25	1.91	0.501	HFGS	5.16	2.32	0.026 *
	M	4.63	2.72		SL	3.82	2.41	
THI	F	52.57	28.43	0.544	HFGS	38.63	20.84	0.011 *
	M	46.33	20.00		SL	58.63	21.65	
SANG	F	54.57	12.04	0.965	HFGS	52.63	11.00	0.207
	M	52.83	9.58		SL	54.32	10.05	
TANG	F	53.00	13.40	0.697	HFGS	52.11	13.49	0.452
	M	54.00	11.75		SL	55.16	10.94	
Cyclothymic	F	4.71	2.95	0.948	HFGS	3.89	2.87	0.013 *
	M	4.75	3.26		SL	5.58	3.19	
Depressive	F	3.86	2.77	0.419	HFGS	3.00	2.73	0.070
	M	3.33	2.44		SL	4.05	2.30	
Irritable	F	2.07	2.16	0.965	HFGS	1.95	2.32	0.359
	M	1.96	1.83		SL	2.05	1.51	
Hyperthymic	F	3.21	1.89	0.004 *	HFGS	3.95	2.25	0.690
	M	4.58	2.22		SL	4.21	2.18	
Anxious	F	1.36	1.28	0.668	HFGS	1.32	1.06	0.663
	M	1.25	1.22		SL	1.26	1.41	
TFI total score	F	58.28	12.12	0.168	HFGS	53.78	15.72	0.693
	M	51.66	15.70		SL	54.41	13.97	
TFI Intrusiveness	F	61.43	10.92	0.745	HFGS	63.33	14.44	0.347
	M	62.36	13.17		SL	60.70	9.79	
TFI Sense of control	F	55.24	17.58	0.604	HFGS	54.91	18.80	0.646
	M	55.83	18.68		SL	56.32	17.74	
TFI Cognitive	F	62.62	13.60	0.073	HFGS	54.39	21.49	0.739
	M	51.39	23.09		SL	56.67	20.31	
TFI Sleep	F	50.24	30.03	0.084	HFGS	39.47	28.74	0.307
	M	39.86	23.56		SL	47.89	23.44	
TFI Auditory	F	63.10	17.81	0.096	HFGS	60.53	17.44	0.452
	M	55.28	16.74		SL	55.79	17.35	
TFI Relaxation	F	59.05	17.99	0.120	HFGS	52.11	21.72	0.998
	M	47.92	23.26		SL	51.93	22.67	
TFI Quality of life	F	57.68	16.27	0.045 *	HFGS	53.25	20.73	0.505
	M	49.03	18.69		SL	51.18	15.58	
TFI Emotional	F	57.38	14.03	0.574	HFGS	52.63	17.59	0.260
	M	52.50	19.22		SL	55.96	17.62	

SD, standard deviation; F, female (N = 14); M, male (N = 24); HFGS, high-frequency gently sloping (N = 19); SL, ski-slope (N = 19); THI, tinnitus handicap inventory; TFI, tinnitus functional index; * statistically significant.

## Data Availability

The data presented in this study are available on request from the corresponding author due to the presence of personal clinical data of the patients included in the study.

## Supplementary Materials

The following supporting information can be downloaded at: https://www.mdpi.com/article/10.3390/medicina62020340/s1, Figure S1: Boxplot illustrating the statistical differences for the gender and audiometric profile from Mann–Whitney U test; Table S1: Correlation analyses of the study variables.

## Abbreviations

The following abbreviations are used in this manuscript:

PTA	Pure tone average
SNHL	Sensorineural hearing loss
STAXI-2	State-Trait Anger Expression Inventory
TEMPS-A	Temperament Evaluation of Memphis, Pisa, Paris, and San Diego—Auto-questionnaire
TFI	Tinnitus functional index
THI	Tinnitus handicap inventory
