# Supplementary material for: Affective Temperaments and Anger in Patients with Tinnitus and High-Frequency Sensorineural Hearing Loss: A Pilot Cross-Sectional Study
Source: Medicina (Kaunas). 2026 Feb 7;62(2):340. doi: 10.3390/medicina62020340 (PMC12942490; doi:10.3390/medicina62020340)
Supplement: Supplementary file 1 [file medicina-62-00340-s001.zip › medicina-4124953-supplementary.pdf]

Figure S1. Boxplot illustrating the statistical differences for the gender and audiometric profile from Mann–Whitney U test.

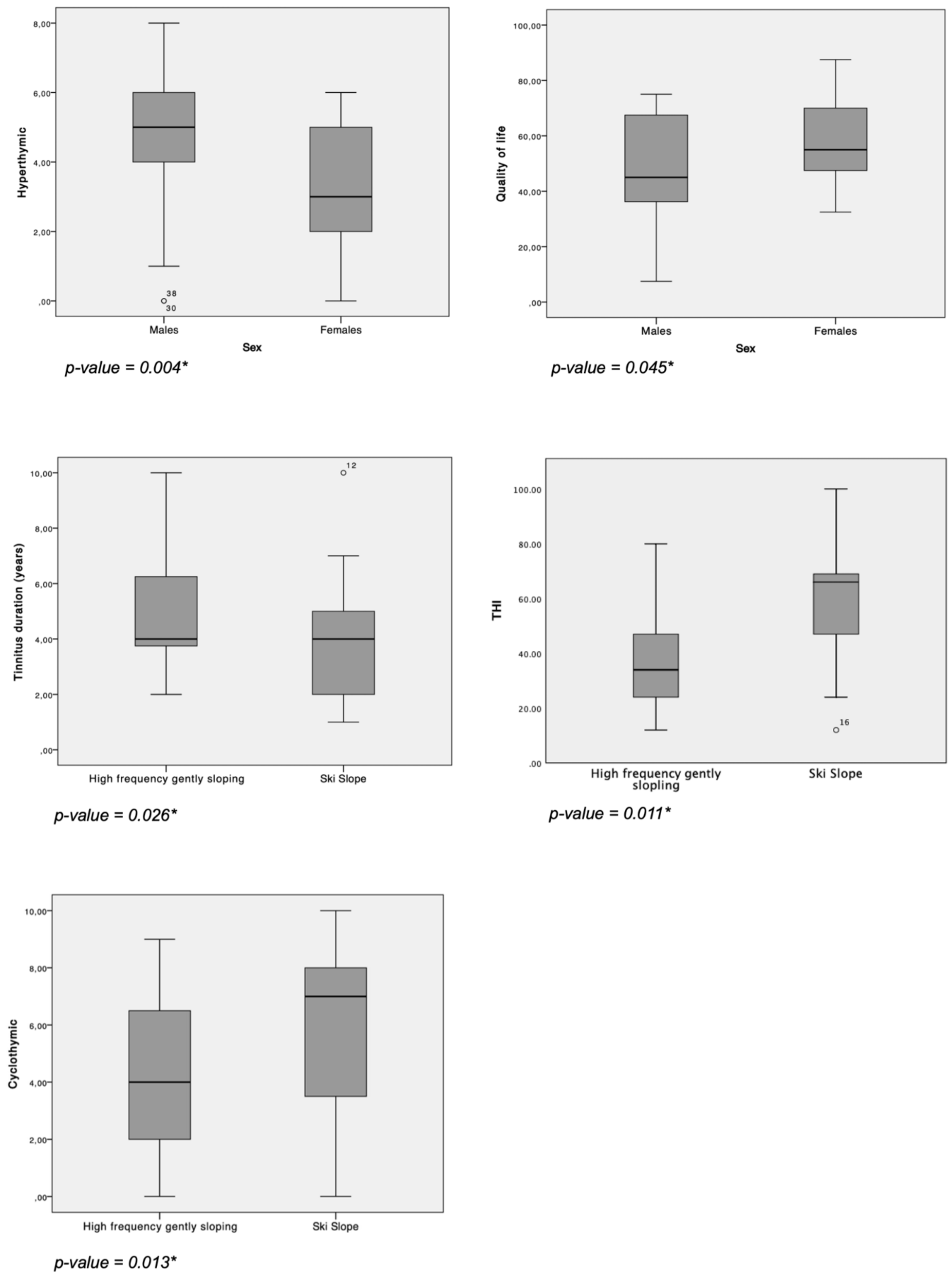

Table S1. Correlation analyses of the study variables.

		1	2	3	4	5	6	7	8	9	10	11	12	13	14	15	16	17
Age (1)	Coeff.	1																
	Signif.	.																
SANG (2)	Coeff.	.021	1															
	Signif.	.901	.															
TANG (3)	Coeff.	-.124	.528**	1														
	Signif.	.460	.001	.														
Cyclothymic (4)	Coeff.	.054	**.495****	**.659****	1													
	Signif.	.747	**.002**	**.000**	.													
Depressive (5)	Coeff.	-.149	**.615****	**.722****	.721**	1												
	Signif.	.371	**.000**	**.000**	.000	.												
Irritable (6)	Coeff.	-.137	**.664****	**.636****	.637**	.796**	1											
	Signif.	.413	**.000**	**.000**	.000	.000	.											
Hyperthymic (7)	Coeff.	.083	**.339***	.016	.167	.154	.202	1										
	Signif.	.622	**.037**	.926	.315	.357	.225	.										
Anxious (8)	Coeff.	.256	.320	.209	.262	.118	.160	.366*	1									
	Signif.	.121	.050	.207	.113	.481	.336	.024	.									
TFI Intrusiveness (9)	Coeff.	.045	-.141	-.064	-.014	-.264	-.149	-.016	-.069	1								
	Signif.	.787	.398	.702	.935	.110	.371	.923	.681	.								
TFI Sense of control (10)	Coeff.	.143	.115	.230	.148	.099	.186	-.086	.141	.407*	1							
	Signif.	.392	.492	.164	.375	.554	.263	.607	.399	.011	.							
TFI Cognitive (11)	Coeff.	.092	.320	.159	.140	.236	.274	.035	.221	.466**	.412*	1						
	Signif.	.582	.050	.340	.400	.153	.096	.835	.182	.003	.010	.						
TFI Sleep (12)	Coeff.	.179	**.533****	.249	.201	.162	.238	.117	**.340***	.241	.382*	.663**	1					
	Signif.	.283	**.001**	.131	.226	.331	.150	.484	**.037**	.145	.018	.000	.					
TFI Auditory (13)	Coeff.	.063	.006	-.001	.075	-.116	-.085	-.002	-.022	.648**	.524**	.427**	.358*	1				
	Signif.	.706	.970	.995	.654	.488	.613	.990	.898	.000	.001	.008	.027	.				
TFI Relaxation (14)	Coeff.	.142	.274	.218	.206	.107	.226	.033	.242	.500**	.512**	.711**	.747**	.690**	1			
	Signif.	.393	.096	.189	.214	.522	.172	.846	.144	.001	.001	.000	.000	.000	.			
TFI Quality of life (15)	Coeff.	.104	.193	.142	.124	.068	.128	-.027	.149	.506**	.468**	.738**	.593**	.722**	.825**	1		
	Signif.	.535	.247	.394	.458	.683	.442	.871	.373	.001	.003	.000	.000	.000	.000	.		
TFI Emotional (16)	Coeff.	.154	.189	.161	.251	.123	.186	-.094	.246	.166	.339*	.488**	.531**	.416**	.565**	.682**	1	
	Signif.	.357	.255	.335	.128	.461	.263	.575	.136	.318	.038	.002	.001	.009	.000	.000	.	
THI (17)	Coeff.	.065	.305	.191	.297	.232	-.021	-.028	**.345***	.219	.232	.272	**.355***	.234	.240	.222	.302	1
	Signif.	.699	.063	.251	.070	.161	.900	.867	**.034**	.187	.161	.098	**.029**	.158	.146	.180	.065	.

** Correlation is significant at the 0.01 level; * Correlation is significant at the 0.05 level.
